# Machine Learning–Based Prediction for Incident Hypertension Based on Regular Health Checkup Data: Derivation and Validation in 2 Independent Nationwide Cohorts in South Korea and Japan

**DOI:** 10.2196/52794

**Published:** 2024-11-05

**Authors:** Seung Ha Hwang, Hayeon Lee, Jun Hyuk Lee, Myeongcheol Lee, Ai Koyanagi, Lee Smith, Sang Youl Rhee, Dong Keon Yon, Jinseok Lee

**Affiliations:** 1 Department of Biomedical Engineering Kyung Hee University Yongin Republic of Korea; 2 Center for Digital Health, Medical Science Research Institute Kyung Hee University College of Medicine Seoul Republic of Korea; 3 Health and Human Science University of Southern California Los Angeles, CA United States; 4 Department of Regulatory Science Kyung Hee University Seoul Republic of Korea; 5 Research and Development Unit Parc Sanitari Sant Joan de Deu Barcelona Spain; 6 Centre for Health, Performance and Wellbeing Anglia Ruskin University Cambridge United Kingdom; 7 Department of Endocrinology and Metabolism Kyung Hee University School of Medicine Seoul Republic of Korea; 8 Department of Pediatrics, Kyung Hee University Medical Center Kyung Hee University College of Medicine Seoul Republic of Korea

**Keywords:** machine learning, hypertension, cardiovascular disease, artificial intelligence, cause of death, cardiovascular risk, predictive analytics

## Abstract

**Background:**

Worldwide, cardiovascular diseases are the primary cause of death, with hypertension as a key contributor. In 2019, cardiovascular diseases led to 17.9 million deaths, predicted to reach 23 million by 2030.

**Objective:**

This study presents a new method to predict hypertension using demographic data, using 6 machine learning models for enhanced reliability and applicability. The goal is to harness artificial intelligence for early and accurate hypertension diagnosis across diverse populations.

**Methods:**

Data from 2 national cohort studies, National Health Insurance Service-National Sample Cohort (South Korea, n=244,814), conducted between 2002 and 2013 were used to train and test machine learning models designed to anticipate incident hypertension within 5 years of a health checkup involving those aged ≥20 years, and Japanese Medical Data Center cohort (Japan, n=1,296,649) were used for extra validation. An ensemble from 6 diverse machine learning models was used to identify the 5 most salient features contributing to hypertension by presenting a feature importance analysis to confirm the contribution of each future.

**Results:**

The Adaptive Boosting and logistic regression ensemble showed superior balanced accuracy (0.812, sensitivity 0.806, specificity 0.818, and area under the receiver operating characteristic curve 0.901). The 5 key hypertension indicators were age, diastolic blood pressure, BMI, systolic blood pressure, and fasting blood glucose. The Japanese Medical Data Center cohort dataset (extra validation set) corroborated these findings (balanced accuracy 0.741 and area under the receiver operating characteristic curve 0.824). The ensemble model was integrated into a public web portal for predicting hypertension onset based on health checkup data.

**Conclusions:**

Comparative evaluation of our machine learning models against classical statistical models across 2 distinct studies emphasized the former’s enhanced stability, generalizability, and reproducibility in predicting hypertension onset.

## Introduction

The World Health Organization (WHO) has identified cardiovascular diseases (CVDs) as the leading cause of mortality worldwide, with a staggering 17.9 million deaths recorded in 2019 [1]. This number is projected to rise to approximately 23 million by 2030. Of the multitude of CVDs, specific conditions such as myocardial infarction and ischemic stroke account for more than 85% of these CVD-related deaths [2]. The US Centers for Disease Control and Prevention (CDC) have highlighted that CVDs caused over US $216 billion in overall health care expenses and resulted in US $147 billion lost due to increased workplace absenteeism and corresponding productivity in the United States. As a result, CVDs impose a significant burden on the nation’s economy [3].

Given the acknowledged biological and economic risks associated with CVDs, it is widely recognized that hypertension plays a significant role in these health complications, including myocardial infarction and stroke [4]. Predicting hypertension onset is notably challenging due to the disease’s multifactorial origins, encompassing a wide range of genetic, environmental, and lifestyle factors. The subtle and often interrelated effects of these factors contribute to the complexity of early detection. For example, genetic predispositions may interact with lifestyle choices such as diet, exercise, and smoking habits, in ways that are not fully understood [5]. Environmental influences, including socioeconomic status and access to health care, further complicate the picture by affecting both the risk of developing hypertension and the ability to manage risk factors effectively [5,6]. Additionally, the asymptomatic nature of hypertension in its early stages means that it often goes unnoticed until more serious health issues arise, making timely and accurate prediction all the more difficult [7]. These challenges underscore the need for sophisticated predictive models that can integrate and analyze the myriad of contributing factors to identify individuals at risk of developing hypertension early in its progression. Considering the severe societal implications of hypertension across all nations, early diagnosis is crucial to mitigate its potential hazards. In this study, we propose a novel approach to predict the onset of hypertension using the population’s regular health checkup and demographic factors. In recent years, machine learning models have emerged as powerful tools across many fields, particularly in medical applications [8]. Their ability to analyze complex patterns and make accurate predictions has revolutionized how we approach health care challenges.

However, ensuring this methodology’s replicability and broad applicability in real-world settings presents an intricate challenge. To bolster the reliability of our hypertension projections, we conducted additional independent validation using distinct cohorts. This study investigated various machine learning approaches to strengthen the method’s robustness, replicability, and real-world practicality. We delved into the hypertension landscape across Asian populations through machine learning optics, firmly anchoring our methodology within the burgeoning realm of artificial intelligence (AI)–driven disciplines. This research endeavors to amplify our comprehension of global hypertension trends by channeling multifaceted machine learning analyses, thereby catalyzing more timely and precise diagnostic efforts.

## Methods

### Data Source

We used 2 national, large-scale, and general population–based cohort studies: the National Health Insurance Service-National Sample Cohort (NHIS-NSC; N=973,303) and the Japanese Medical Data Center cohort (JMDC; N=12,143,715). This study was approved by the institutional review board of National Health Insurance Service, Kyung Hee University (KHSIRB-23-085[EA]), and the JMDC (PHP-00002201-04). The requirement for informed consent was waived as this study used deidentified administrative data.

### NHIS-NSC (Discovery Cohort)

The NHIS-NSC [9], the population-based, nationwide, and large-scale cohort of South Korea, were from those aged ≥20 years who received general health checkups between January 1, 2002, and December 31, 2013. We used the NHIS-NSC to train, validate, and test the machine learning model to predict the presence or absence of hypertension within 5 years of a regular (yearly) health checkup. Hypertension was defined for patients who had received diagnoses with I10, I11, I12, I13, or I15 codes from the *ICD-10* (*International Classification of Disease, 10th revision*) ≥2 times and were using antihypertensives [10].

During the data preprocessing phase, we transformed the cohort into a machine learning dataset by representing each eligible individual once, with all features recorded from their initial health check-up. The ground truth was determined by the occurrence of a hypertension event within the subsequent 5 years. We excluded participants with baseline hypertension or those lost to follow-up from this study. Individuals who developed hypertension after 5 years were classified as nonhypertensive for this study.

**Figure 1:**
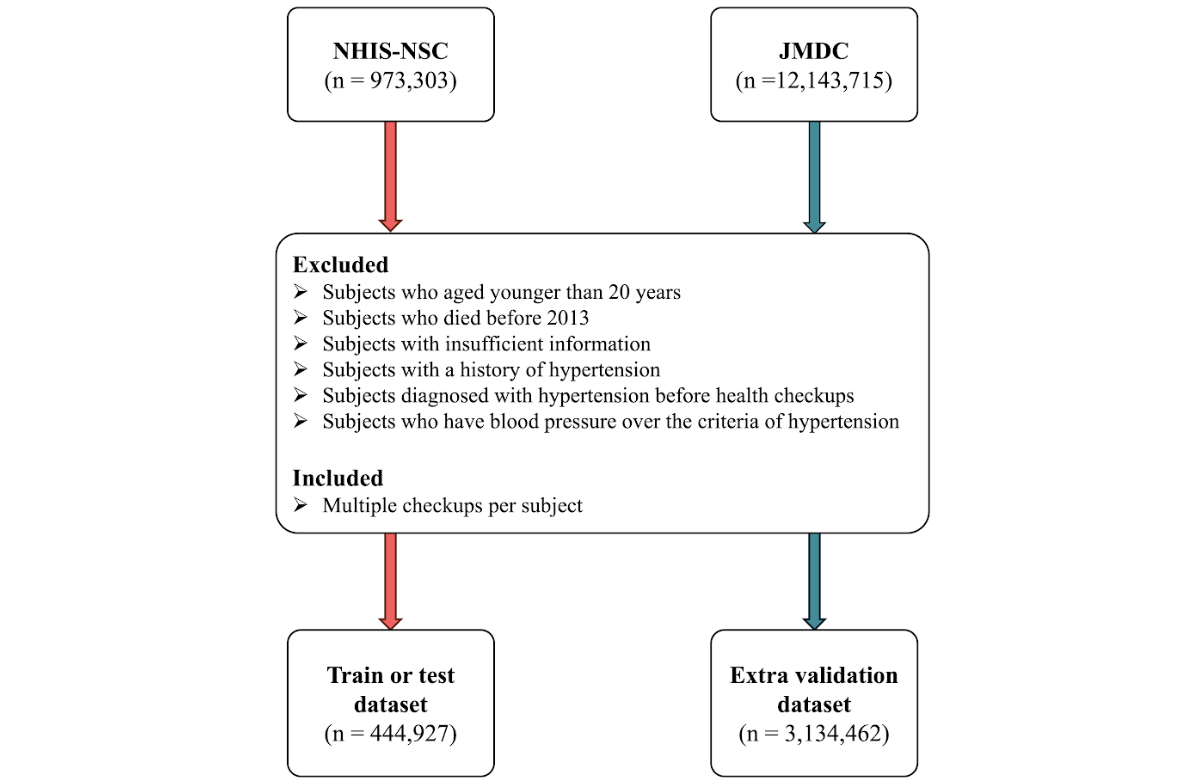
Study population and data selection process in the NHIS-NSC (Korea) and JMDC (Japan). NHIS-NSC: National Health Insurance Service-National Sample Cohort; JMDC: Japanese Medical Data Center cohort.

In this study, we excluded participant information that fulfilled one of the following criteria among the 973,303 registered participants: (1) those who had reported “yes” for hypertension in the questionnaire; (2) those who had a prior diagnosis of hypertension with I10, I11, I12, I13, or I15 codes of *ICD-10* before the health checkup; (3) those with missing data for information and questionnaire; (4) those who had died before the year 2013; and (5) those who have blood pressure over the criteria of hypertension (systolic blood pressure ≥140 mm Hg or the diastolic blood pressure is ≥90 mm Hg). A graphical representation of the subject exclusion process of the NHIS-NSC is illustrated in Figure 1 [11].

### JMDC (Validation Cohort)

The JMDC dataset is the medical examination data from multiple health insurance associations in Japan since 2005 [12-14]. Given the machine learning model trained from the NHIS-NSC, we used the JMDC data for extra validation. We also applied the same exclusion criteria used in the NHIS-NSC to the JMDC data, resulting in the use of only 1,296,649 participant data of the total 12,143,715 data available. A graphical representation of the subject exclusion process of the JMDC dataset is also illustrated in Figure 1.

### Study Design

To develop the machine learning model for predicting the presence or absence of hypertension within 5 years of a regular (yearly) health checkup, we used the following 18 available variables as the model’s input: age, sex, region of residence, household income, systolic blood pressure, diastolic blood pressure, fasting blood glucose, serum total cholesterol, hemoglobin, aspartate transaminase (AST), alanine transaminase (ALT), γ-glutamyl transpeptidase (γ-GTP), BMI, history of diabetes mellitus, history of stroke (including ischemic stroke, hemorrhagic stroke, and/or transient ischemic attack), smoking status, alcohol intake, and physical activity [15]. The variables used in our machine learning model are summarized in Table 1. More specifically, the region of residence was categorized into rural and urban. Household income was categorized into 11 scales (0 to 10) based on basic livelihood recipient and decile (Table S1 in Multimedia Appendix 1); in 10 income deciles, the 5th decile is the reference median income. Compared to 5th decile (100%), 1st decile (the lowest income level) has an income of less than 30%, 2nd decile has an income of less than 50%, 3rd decile has an income of less than 70%, 4th decile has an income of less than 90%, 6th decile has an income of less than 130%, 7th decile has an income of less than 150%, 8th decile has an income of less than 200%, 9th decile has an income of less than 300%, and 10th decile (the highest income level) has an income of 300% or more. Basic livelihood recipients are individuals whose income falls within 1st decile (the lowest 30%) [12]. Smoking status was categorized into never, former, and current smokers. Alcoholic intakes were categorized into rare (less than one time per week), 1-2, 3-4, and more than four times per week [12-14]. Physical activity was categorized into never, 1-2, 3-4, and 5-6 times per week, and every day. The statistical characteristics of the variables for the NHIS-NSC and JMDC are summarized in Tables 1 and 2, respectively.

**Table 1:** Baseline characteristics of subjects in the discovery cohort (National Health Insurance Service-National Sample Cohort, N=244,814).

Variables			Values
**Sex, n (%)**			
	Male		117,642 (48.05)
	Female		127,172 (51.95)
Age (years), mean (SD)			47.03 (13.29)
**Region of residence, n (%)**			
	Urban		111,640 (45.6)
	Rural		133,174 (54.4)
**Household income, n (%)**			
	Basic livelihood recipient		437 (0.18)
	**Income deciles (excluded basic livelihood recipients)**		
		D1 (the lowest income level; ≤30th percentile)	18,783 (7.67)
		D2 (31st-50th percentile)	19,399 (7.92)
		D3 (51st-70th percentile)	22,165 (9.05)
		D4 (71st-90th percentile)	24,498 (10.01)
		D5 (91st-100th percentile)	25,043 (10.23)
		D6 (101st-130th percentile)	26,137 (10.68)
		D7 (131st-150th percentile)	26,195 (10.7)
		D8 (151st-200th percentile)	26,562 (10.85)
		D9 (201st-300th percentile)	28,469 (11.63)
		D10 (high income level, >300th percentile)	27,126 (11.08)
Systolic blood pressure (mm Hg), mean (SD)			111.6 (9.42)
Diastolic blood pressure (mm Hg), mean (SD)			68.69 (6.1)
Fasting blood glucose (mg/dL), mean (SD)			92 (23.48)
Serum total cholesterol (mg/dL), mean (SD)			187.91 (35.84)
Hemoglobin (g/dL), mean (SD)			13.78 (1.58)
Aspartate transaminase (U/L), mean (SD)			23.82 (15.12)
Alanine transaminase (U/L), mean (SD)			22.9 (21.32)
γ-glutamyl transpeptidase (U/L), mean (SD)			28.81 (37.6)
BMI (kg/m^2^), mean (SD)			22.8 (3)
History of diabetes mellitus, n (%)			4596 (1.88)
History of stroke, n (%)			376 (0.15)
**Smoking status, n (%)**			
	Nonsmoker		176,333 (72.03)
	Ex-smoker		9148 (3.74)
	Current smoker		59,333 (24.24)
**Alcohol intake per week, n (%)**			
	Rarely		182,101 (74.38)
	1-2		45,374 (18.53)
	3-4		12,337 (5.04)
	≥5		5002 (2.04)
**Physical activity per week, n (%)**			
	Never		141,847 (57.94)
	1-2		63,046 (25.75)
	3-4		23,426 (9.57)
	5-6		5828 (2.38)
	Every day		10,667 (4.36)

**Table 2:** Baseline characteristics of subjects in the validation cohort (Japanese Medical Data Center cohort; N=1,296,649).

Variables		Values
**Sex, n (%)**		
	Male	754,055 (58.15)
	Female	542,594 (41.85)
Age (years), mean (SD)		42.51 (10.24)
Systolic blood pressure (mm Hg), mean (SD)		111.65 (10.43)
Diastolic blood pressure (mm Hg), mean (SD)		67.88 (7.55)
Fasting blood glucose (mg/dL), mean (SD)		91.45 (14.31)
Serum total cholesterol (mg/dL), mean (SD)		200.05 (35.25)
Hemoglobin (g/dL), mean (SD)		14.19 (1.56)
Aspartate transaminase (U/L), mean (SD)		20.84 (8.89)
Alanine transaminase (U/L), mean (SD)		21.02 (15.86)
γ-glutamyl transpeptidase (U/L), mean (SD)		30.63 (33.48)
BMI (kg/m^2^), mean (SD)		22.12 (3.22)
History of diabetes mellitus, n (%)		14,345 (1.11)
History of stroke, n (%)		3616 (0.28)
**Smoking, n (%)**		
	No	978,245 (75.44)
	Yes	318,404 (24.56)
**Alcohol intake per week, n (%)**		
	Rarely	669,090 (51.6)
	Sometimes	403,527 (31.12)
	Every day	224,052 (17.28)
**Physical activity, n (%)**		
	No	1,082,572 (83.49)
	Yes	214,077 (16.51)

### Proposed Machine Learning Models

In this study, we split the NHIS-NSC dataset (n=244,814) into train (n=195,851) and internal test (n=48,963) data with a ratio of 8:2 in a stratified fashion. The internal test set was used only for an independent test of our developed AI model and not for training or internal validation. The JMDC (n=1,296,649) was used as the external validation dataset in this study.

The data distribution was severely imbalanced: the ratio of hypertension and nonhypertension group was 1:15.32. To minimize the bias toward the majority group (nonhypertension) of the prediction model, we up-sampled the hypertension data using a synthetic minority oversampling technique during the model update [16]. In addition, in the preprocessing stage, we performed standard scaler normalization for all features: we calculated the mean and SD of each feature from the training dataset and then normalized all feature values from both the test dataset and external validation datasets to have a mean of 0 and a SD of 1.

To predict hypertension occurrence within 5 years based on regular health check-ups, we applied 6 machine learning models from 18 features: Extreme Gradient Boosting, random forest, gradient boosting machine (GBM), Light GBM, Adaptive Boosting (AdaBoost), and logistic regression (LR) [17,18]. Subsequently, we chose the best 3 among the 6 models and applied an ensemble approach by considering all possible combinations [19]. Performance evaluations were based on 5-fold cross-validation using the train data following metrics: sensitivity, specificity, accuracy, balanced accuracy, and area under the receiver operating characteristics (AUROC) [17,18]. To compare the predictive performance of the models, we performed a Cochrane Q test on the model performance [20,21]. Due to the significant data imbalance, we used balanced accuracy as the primary model evaluation metric. Moreover, we also estimated additional metrics to comprehensively evaluate the performance of each model: precision, *F*_1_-score, and area under the precision-recall curve. To address the issue of inappropriate precision and *F*_1_-score under the severe data imbalance, we measured weighted average precision and weighted average *F*_1_-score, accounting for the differences in class sizes. Finally, we presented its feature importance analysis, listing features in the order they contributed to hypertension prediction within 5 years of regular health checkups.

We implemented the models using Python (version 3.9.16; Python Software Foundation) with TensorFlow (version 2.9.1; Google LLC), Keras (version 2.9.0; Google LLC), NumPy (version 1.21.5; NumFOCUS, Inc), Pandas (version 1.4.4; NumFOCUS, Inc), Matplotlib (version 3.5.2; NumFOCUS, Inc), and Scikit-learn (version 1.0.2; NumFOCUS, Inc) [18,22]. All statistical analysis was performed using SAS (version 9.4, SAS Institute Inc) [22].

### Feature Importance

To analyze the effect of each feature on predicting hypertension occurrence, we performed the feature importance analysis to confirm the contribution of each feature. For tree-based models, the mean decrease in impurity (MDI), which is also known as Gini importance, is used to assess feature importance [23,24]. The following equation represents MDI [24]:



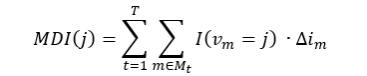





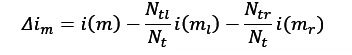



where *T* is the total number of trees in the base estimator, is the set of all nodes in tree *t*, is the feature used for splitting the node *m*, ​ is the decrease in impurity at node *m*, is the Gini impurity, is the number of samples at node *m*, and is an indicator function that is 1 if feature *j* is used for splitting at node *m* and 0 otherwise. Notation of *l* and *r* indicates left child node and right child node each. Those equations calculate feature importance by summing up the impurity reductions caused by each feature across all trees in the model. A higher MDI indicates greater feature importance. For LR, we used the regression coefficient to calculate feature importance. The following equation indicates the LR model [25]:



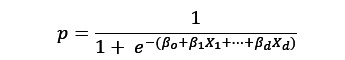





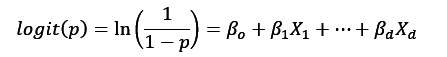



where *d* is the number of features. The regression coefficient describes the average change in the dependent variable for each 1-unit change in the independent variable for continuous independent variables or the expected difference versus a reference category for categorical independent variables. Further, for ensembled models, we calculated feature importance by averaging the standardized feature importance from each model used for the ensemble.

### Risk Factors

We further investigated the association between the occurrence of hypertension and independent variables using univariate and multivariate LR analyses in the discovery and validation cohorts [26,27]. Predictor variables included categorical variables (sex, region of residence, history of diseases, smoking status, alcohol intake, and physical activity) and continuous variables transformed into categorical form (age, household income, BMI, blood pressure, fasting blood glucose, serum total cholesterol, hemoglobin, ALT, AST, and γ-GTP). Univariable and multivariable LR analyses were conducted for each variable to estimate the odds ratio and 95% CI for the occurrence of hypertension. All statistical analyses were conducted using SAS (version 9.4, SAS Institute) [28].

### Ethical Considerations

The claims-based cohort data in South Korea and Japan were anonymous, and this study’s protocol was approved by the Institutional Review Board of National Health Insurance Service, Kyung Hee University (KHSIRB-23-085(EA)), and the JMDC (PHP-00002201-04).

## Results

### K-Fold Cross-Validation

For the 6 machine learning models, we found the following optimized hyper-parameters using grid search with 5-fold cross-validation: For Extreme Gradient Boosting, we used booster type of gradient boosted tree, column subsample by tree 0.1, learning rate 0.2, maximum depth of 3, and number of estimators 100. For random forest, we used maximum depth of 3, maximum features of 3, minimum samples per leaf 3, minimum samples per split 3, number of estimators 50, and balanced class weight. For Light GBM, we used boosting parameter of gradient-based 1-sided sampling, objective function of binary classification objective, evaluation metrics of log loss function for binary classification, learning rate 0.002, number of estimators 70, and number of leaves 30. For GBM, we used learning rate 0.008, maximum depth of 2, minimum samples per leaf 3, minimum samples per split 3, and number of estimators 100. For AdaBoost, we used algorithm of stagewise additive modeling using a multiclass exponential loss function, real variant; number of tree estimators with 500; and learning rate with 0.02. For LR, the solver of the library for large linear classification; the penalty norm was with L2, inverse of regularization strength 0.1, and the maximum number of iterations was with 100. For ensemble models, we used the same hyperparameters as those used in the individual machine learning models. Additionally, no weights were applied when combining the models in the ensemble. After finding the optimal hyperparameters, we checked the performance of each model and ensemble model. To improve performance, we tuned the models to use optimal thresholds through AUROC [20,29]. The optimized threshold values for some models are as follows: 0.48 for AdaBoost, 0.46 for GBM and AdaBoost, 0.46 for LR and GBM, and 0.46 for the GBM, AdaBoost, and LR. Table 3 summarizes the 5-fold cross-validation accuracy comparison of each model and ensemble machine learning models using sensitivity, specificity, accuracy, balanced accuracy, and AUROC as evaluation metrics. Among the single models, GBM, LR, and AdaBoost demonstrated the best prediction performance per balanced accuracy and AUROC. To further improve the classification performance, we explored an ensemble approach using the top-3 single models: GBM, LR, and AdaBoost. The results show that the combination of LR and AdaBoost provides the highest performance with a sensitivity of 80.62%, specificity of 81.79%, balanced accuracy of 81.2%, and AUROC of 0.9012. In addition, we also summarize 3 additional metrics suitable for imbalanced data in Table S2 in Multimedia Appendix 1: weighted average precision, weighted average *F*_1_-score, and area under the precision-recall curve.

**Table 3:** Comparison of the prediction performances of the prediction models on the training dataset in the discovery cohort^a^.

Model	Sensitivity, mean (SD)	Specificity, mean (SD)	Accuracy, mean (SD)	Balanced accuracy, mean (SD)	AUROC^b^, mean (SD)	*P* values^c^
AdaBoost^d^	0.8503 (0.0074)	0.7725 (0.0048)	0.7764 (0.0044)	0.8114 (0.0023)	0.9136 (0.0035)	<.001
LR^e^	0.8009 (0.0090)	0.8076 (0.0015)	0.8072 (0.0012)	0.8042 (0.0041)	0.8819 (0.0046)	<.001
XGBoost^f^	0.6208 (0.011)	0.9599 (0.0029)	0.943 (0.0029)	0.7904 (0.006)	0.8866 (0.0052)	<.001
Random forest	0.7328 (0.012)	0.8642 (0.0098)	0.8577 (0.009)	0.7985 (0.0052)	0.8875 (0.0056)	<.001
Light GBM^g^	0.8295 (0.0052)	0.7649 (0.0033)	0.7681 (0.0032)	0.7972 (0.0038)	0.8743 (0.0058)	<.001
GBM	0.7853 (0.0065)	0.8194 (0.0027)	0.8176 (0.0029)	0.8023 (0.0046)	0.8942 (0.0051)	<.001
GBM and AdaBoost	0.8194 (0.0063)	0.8006 (0.0065)	0.8016 (0.0061)	0.81 (0.0035)	0.9063 (0.0044)	<.001
LR and GBM	0.8221 (0.0081)	0.7934 (0.0012)	0.7949 (0.0012)	0.8078 (0.0041)	0.9009 (0.0048)	<.001
GBM, AdaBoost, and LR	0.8373 (0.0076)	0.7795 (0.0014)	0.7824 (0.0011)	0.8084 (0.0034)	0.9065 (0.0047)	<.001
AdaBoost and LR	0.8062 (0.0072)^h^	0.8179 (0.0015) ^h^	0.8173 (0.0012) ^h^	0.8120 (0.0030) ^h^	0.9012 (0.0046) ^h^	Reference

^a^All outcomes are averaged over 5-fold cross-validation.

^b^AUROC: area under receiver operating characteristic.

^c^To compare the predictive performance of the models, we performed a Cochrane Q test on the model performance.

^d^AdaBoost: Adaptive Boosting.

^e^LR: logistic regression.

^f^XGBoost: Extreme Gradient Boosting.

^g^GBM: gradient boosting machine.

^h^Indicates machine learning model with best performance of prediction.

### Feature Importance Analysis

The ranked normalized feature importance is from the ensemble model combining AdaBoost and LR. According to the results, age had the highest importance value among the features, followed by diastolic blood pressure, BMI, systolic blood pressure, and fasting blood glucose. Feature importances are as follows: age, 1.00; diastolic blood pressure, 0.93; BMI, 0.75; systolic blood pressure, 0.58; fasting blood glucose, 0.35; γ-GTP, 0.24; serum total cholesterol, 0.18; ALT, 0.10; AST, 0.097; history of diabetes mellitus, 0.087; household income, 0.77; hemoglobin, 0.025; sex, 0.021; history of stroke, 0.014; physical activity, 0.010; alcohol intake per week, 0.0077; region of residence, 0.0065; and smoking, 0.0055.

### Ablation Study

Table S3 in Multimedia Appendix 1 summarizes the ablation study results when one or some top 5-contribution features were excluded: age, diastolic blood pressure, BMI, systolic blood pressure, and fasting blood glucose. Without age, the model provides poor prediction performance: balanced accuracy dropped from 0.812 to 0.782, and AUROC dropped from 0.901 to 0.864. Without diastolic blood pressure, balanced accuracy dropped to 0.784, and AUROC dropped to 0.871. Without BMI, balanced accuracy dropped to 0.811, and AUROC dropped to 0.898. Without systolic blood pressure, balanced accuracy dropped to 0.801, and AUROC dropped to 0.890. Without fasting blood glucose, balanced accuracy dropped to 0.8118, and AUROC dropped to 0.9010. Additionally, we analyzed the model performance when the 2 blood pressure features (systolic and diastolic) were excluded. The results show that the performance significantly degraded across all accuracy metrics: balanced accuracy from 0.812 to 0.725 and AUROC from 0.901 to 0.797.

### Test Data Results and External Validation Results

Table 4 summarizes the test data results from the test dataset from the NHIS-NSC and the external validation data results from the JMDC. The test data results also showed that the ensemble model combining AdaBoost and LR provides the highest value of balanced accuracy (0.8147). The similarity between the cross-validation and test data results denotes minimal overfitting or underfitting. The external validation data results also showed the ensemble model combining AdaBoost and LR provided the highest value of balanced accuracy (0.7406). The results confirmed that our ensemble model combining AdaBoost and LR could provide an accurate prediction of hypertension within 5 years based on the regular health checkup data.

**Table 4:** Comparison of the prediction performances of the prediction models on the test dataset (discovery cohort) and the external validation dataset (validation cohort).

Model		Sensitivity	Specificity	Accuracy	Balanced accuracy	AUROC^a^
**Test dataset (discovery cohort)**						
	AdaBoost^b^	0.8573	0.7677	0.7722	0.8125	0.9123
	LR^c^	0.8093	0.8064	0.8066	0.8078	0.8832
	XGBoost^d^	0.6257	0.9629	0.9461	0.7943	0.8886
	Random forest	0.8913	0.6417	0.6542	0.7665	0.8598
	Light GBM^e^	0.8230	0.7707	0.7733	0.7968	0.8714
	GBM	0.7839	0.8379	0.8352	0.8109	0.8966
	GBM and AdaBoost	0.8403	0.7884	0.7910	0.8143	0.9080
	LR and GBM	0.8297	0.7988	0.8004	0.8143	0.9039
	GBM, AdaBoost, and LR	0.8443	0.7841	0.7871	0.8142	0.9087
	AdaBoost and LR	0.8129^f^	0.8165^f^	0.8163^f^	0.8147^f^	0.9022^f^
**External validation dataset (validation cohort)**						
	AdaBoost	0.6724	0.7906	0.7840	0.7315	0.8148
	LR	0.6378	0.8352	0.8242	0.7365	0.8134
	XGBoost	0.5253	0.8724	0.8530	0.6989	0.7906
	Random forest	0.7109	0.6715	0.6737	0.6912	0.7324
	Light GBM	0.5446	0.8241	0.8084	0.6843	0.7402
	GBM	0.4995	0.8869	0.8652	0.6932	0.7875
	GBM and AdaBoost	0.5906	0.8349	0.8212	0.7127	0.8052
	LR and GBM	0.6428	0.8379	0.8270	0.7404	0.8241
	GBM, AdaBoost, and LR	0.6240	0.8546	0.8417	0.7393	0.8271
	AdaBoost and LR	0.6354^f^	0.8458^f^	0.8341^f^	0.7406^f^	0.8242^f^

^a^AUROC: area under receiver operating characteristic.

^b^AdaBoost: Adaptive Boosting.

^c^LR: logistic regression.

^d^XGBoost: Extreme Gradient Boosting.

^e^GBM: gradient boosting machine.

^f^Indicates machine learning model with best performance of prediction.

### Association Between Risk Factors and Occurrence of Hypertension

The association between the occurrence of hypertension and potential risk factors is presented in Tables S4 and S5 in Multimedia Appendix 1. In both the discovery and validation cohorts, consistently, the multivariable model revealed that older age, female sex, urban residence, high income, high blood pressure, high serum total cholesterol, high hemoglobin, high AST, high γ-GTP, high BMI, history of diabetes mellitus, history of stroke, frequent alcohol intake, and insufficient physical activity were significantly associated with an increased risk of hypertension.

### AI-Driven Web Application

Our proposed ensemble model was deployed on our own public website [30] so that hypertension onset within 5 years can be predicted based on regular health checkup data. The deployed web application, which provides results for prediction of hypertension onset, is shown in Figure S1 in Multimedia Appendix 2. The web interface for entering information on 18 features from regular health checkup data is shown in Figure S1(a) in Multimedia Appendix 2. After entering the information in the web application, a user can immediately obtain the results for prediction of hypertension onset with its probability, as shown in Figure S1(b) in Multimedia Appendix 2. In the web application, the features input by a user are encoded to the website server, and immediately deleted upon generation of the prediction result, so that there is no risk of exposing information. In addition, there is no need to enter any information that would be regarded as private. Furthermore, we have open-sourced the Python code for the proposed ensemble model as publicly available in a GitHub repository [31].

## Discussion

### Main Findings

Given the significant health and economic consequences of CVDs, particularly myocardial infarction and stroke, it is essential to examine hypertension, a principal contributing factor to these conditions. This study uses data from 244,814 South Korean participants, obtained from the NHIS-NSC over a 12-year study period, and data from 1,296,649 Japanese participants, collected by the JMDC from various health insurance associations in Japan since 2005.

Our findings indicated that an ensemble of AdaBoost and LR models provided superior performance, achieving a sensitivity of 80.62%, specificity of 81.79%, balanced accuracy of 81.2%, and AUROC of 90.12%, suggesting that quantifying the occurrence of hypertension using feature importance analysis with ensemble machine learning (AdaBoost and LR) can enhance generalizability and reproducibility.

Using our knowledge of the machine learning model, our study has analyzed the occurrences of hypertension. Using feature importance analysis, our study has indicated the top 5-contribution features of hypertension, which were age, diastolic blood pressure, BMI, systolic blood pressure, and fasting blood glucose. Following the feature importance analysis, to measure the impact of such contributing features of hypertension, through an ablation study, we have excluded some contribution features among the top 5 contributing features of hypertension.

From our further investigation into the association between hypertension and independent variables, we analyzed various risk factors. Our analysis revealed that older age, female sex, urban residence, high income, elevated blood pressure, high serum total cholesterol, elevated hemoglobin, high AST, high γ-GTP, high BMI, history of diabetes mellitus, history of stroke, frequent alcohol intake, and insufficient physical activity were significantly associated with an increased risk of hypertension.

After obtaining test data results, through extra validation using the JMDC dataset, we have validated that our ensemble model combining AdaBoost and LR could provide an accurate prediction of hypertension within 5 years based on the regular health checkup data (balanced accuracy 0.741 and AUROC 0.824). Using such analysis of both the NHIS-NSC and JMDC as original and extra validation, our study has established a web application allowing diagnosis of hypertension [32-34].

### Comparison With Previous Studies

Similar to our study, past research efforts have worked on developing hypertension risk prediction models using variables akin to our study, including age, sex, BMI, blood pressure metrics, parental hypertension history, smoking habits, and in certain cases, additional markers such as C-reactive protein, apolipoprotein A, and uric acid [35] (United States, n=1717 [36], n=1130 [37], n=15,732 [38], n=876 [39], and n=23,095 [40]; United Kingdom, n=10,308 [41]; and Iran, n=380 [42]).

Although there were several prior studies to find the occurrence of hypertension and establish web applications, many of these studies presented limitations, presenting problems such as producing low levels of reliability and yielding conflicting results. These constraints can be attributed to smaller sample sizes, short follow-up durations, and inadequate study designs such as nonrepresentative or nonrandom selection of populations [37,40,42]. Additionally, most of the studies have not held web application-fortifying processes, such as extra validation studies.

Our research stands apart in this context. We used a longitudinal approach using extensive datasets from both South Korean and Japanese health insurance databases, encompassing a comprehensive range of hypertension-related data spanning over 10 years. By leveraging datasets from 244,814 individuals in South Korea and 1,296,649 in Japan, we implemented a 5-fold cross-validation for optimizing an ensemble machine learning model. This was followed by a feature importance analysis to identify the top 5 determinants of hypertension, an ablation study to gauge the significance of each contributing factor, and an additional validation procedure. As a result, our work culminated in the development of a robust machine learning-powered web application, a milestone that many preceding studies fell short of achieving.

### Possible Explanations for Our Results

This study harnesses real-world data where conventional statistical methods often struggle to guarantee generalizability and reproducibility in real-life situations. However, such challenges can be surmounted with AI-powered machine learning techniques such as variable pruning and group optimization.

By integrating machine learning methodologies, specifically AdaBoost and LR, our research can perpetually evaluate potential features linked to hypertension onset. This translates to a resilient system adept at observing the correlation between standardized traits and hypertension episodes, which include age, diastolic blood pressure, BMI, systolic blood pressure, and fasting blood glucose concentrations. Notably, this strategy offers considerable benefits, ensuring dependable data on hypertension prevalence across a wide demographic, even if the analysis encompasses merely a fraction of the overall populace. Additionally, our pioneering methodology offers a distinct advantage by ensuring accessibility even for individuals who may be illiterate or disinclined to participate in hypertension-specific screenings. This capacity for rapid diagnostic evaluation equips health care professionals with the tools to offer more targeted and accurate services to patients facing hypertension risks.

### Policy Implication

Our diagnostic method’s validation, achieved via an ensemble machine learning strategy integrating AdaBoost and LR, consistently upholds accuracy in hypertension identification, even among newly discerned populations potentially susceptible to hypertension. This tool not only paves the way for preemptive hypertension identification but also extends its reach to individuals distant from conventional health care infrastructure, such as hospitals and regional health centers. Our study is keen on transitioning our web-based platform to a mobile app [18], addressing and eliminating any accessibility barriers. Such an evolution positions our tool as a universally accessible resource, irrespective of an individual’s socioeconomic status, domicile, or the developmental index of their nation. Worldwide, national administrations can advocate for our tool, empowering citizens to independently gauge their hypertension risk and pursue timely medical interventions. The distinct advantage of our platform is its avoidance of potential diplomatic sensitivities, given its nonreliance on any personal or confidential data.

### Strengths and Limitations

An astute examination of this study’s findings calls for recognizing inherent limitations. To elaborate, even though our research draws on data from 2 distinct cohorts—the NHIS-NSC (n=244,814) for training or testing and the JMDC (n=1,296,649) for extra-validation—these datasets encompass but a marginal segment of the overarching Asian demographic, and an even lesser representation of the worldwide populace. This fact accentuates the imperative for our conclusions to be subjected to broader international validation studies and exhaustive longitudinal investigations. Furthermore, although the sample size of the JMDC is larger, its somewhat limited set of variables led us to develop a model using the comprehensive set of variables available in the NHIS-NSC [43]. Despite the smaller size of the NHIS-NSC, our proposed ensemble model showed stable and consistent performance when validated with the JMDC. Moreover, it is crucial to acknowledge that our study tested a limited array of model types, excluding machine learning models such as k-nearest neighbors and support vector machines. Including these models would have provided a comprehensive comparison and potentially strengthened our findings. Additionally, our analysis did not include certain hypertension-related features, such as family history, dietary habits, and salt consumption. As it is well known that these factors play a significant role in the development and progression of hypertension, their absence may have influenced the predictive power of the models and the holistic understanding of hypertension risk factors. Lastly, segmenting related variables such as systolic and diastolic pressure, ALT, and AST can capture diverse aspects and reduce data loss, but it may dilute significance due to their correlation [44].

While the primary aim of our study has been to identify predictors for the onset of hypertension, we acknowledge that predicting the magnitude of blood pressure increases offers an invaluable perspective on the complex interplay between initial blood pressure levels and their changes over time. This area, although not explored within the current scope of our research, holds significant potential for advancing our understanding of hypertension. Future investigations that include baseline blood pressure measurements could yield profound insights into the risk factors and dynamics of blood pressure changes. Such research would enrich our predictive models and refine management strategies for hypertension, marking a crucial step forward in the field.

Yet, amid these confines, one must not undermine this study’s significance. Our endeavor capitalizes on data meticulously gathered for over a decade from South Korea and Japan. In a methodical exercise of comparing a spectrum of 6 machine learning models and subsequently analyzing ensemble variations, we astutely pinpointed the critical determinants closely aligned with hypertension onset, ensuring commendable reproducibility and applicability. Furthermore, the genesis of a user-responsive web tool, facilitating individuals to input personal health metrics, epitomizes our groundbreaking stride toward expeditious, precision-driven, and worldwide accessible diagnostic avenues for hypertension.

### Conclusions

In a pioneering endeavor, this research uniquely integrates both machine learning and conventional statistical frameworks to prognosticate the emergence of hypertension. A notable outcome of this exploration is the institution of a digital platform adept at forecasting a 5-year onset of hypertension, using data sourced from the NHIS-NSC and JMDC. Our empirical outcomes, extrapolated from 2 autonomous studies, substantiate that machine learning paradigms—particularly an amalgamation of AdaBoost and LR—eclipsed the traditional statistical methodologies in preempting hypertension onset. A meticulous inquiry was undertaken to ascertain the hierarchical significance of determinants linked with hypertension. The investigation earmarked age as the paramount factor, trailed by diastolic blood pressure, BMI, systolic blood pressure, and fasting blood glucose concentrations. This sequence, pivotal in curating the most efficacious machine learning model and subsequent hypertension emergence, was further corroborated via supplementary validation harnessing the JMDC datasets. Emerging from these discernments is an AI-infused digital interface, proficient in envisioning a quintennial likelihood of hypertension based on routine health assessment metrics. Such an innovation positions itself as an instrumental diagnostic conduit for individuals predisposed to hypertension.

## Appendix

Multimedia Appendix 1 Sensitivity analysis.

Multimedia Appendix 2 AI-driven web application. AI: artificial intelligence.

## Abbreviations

AI: artificial intelligence
ALT: alanine transaminase
AST: aspartate transaminase
AUROC: area under the receiver operating characteristic
CDC: Centers for Disease Control and Prevention
CVD: cardiovascular disease
GBM: gradient boosting machine
ICD-10: International Classification of Disease, 10th revision
JMDC: Japanese Medical Data Center cohort
LR: logistic regression
MDI: mean decrease in impurity
NHIS-NSC: National Health Insurance Service-National Sample Cohort
WHO: World Health Organization
γ-GTP: γ-glutamyl transpeptidase

## Data Availability

Data are available on reasonable request to the corresponding authors and with appropriate ethical approvals.
