# Supplementary material for: Machine Learning–Based Prediction for Incident Hypertension Based on Regular Health Checkup Data: Derivation and Validation in 2 Independent Nationwide Cohorts in South Korea and Japan
Source: J Med Internet Res. 2024 Nov 5;26:e52794. doi: 10.2196/52794 (PMC11576616; doi:10.2196/52794)
Supplement: Multimedia Appendix 1 [file jmir_v26i1e52794_app1.docx]

**Table S1.** The calculation criteria for household income (as of 2024; with an exchange rate of approximately US $1 ≈ KR ₩1350).

|  | **Household income (per month)** | **Standard median household income ratio** |
| --- | --- | --- |
| 0 (basic livelihood recipient) | ≤2,291,965 KRW (1698 USD) with medical assistance beneficiary | 40% |
| 1 | ≤1,718,974 KRW (1273 USD) | 30% |
| 2 | ≤2,864,957 KRW (2122 USD) | 50% |
| 3 | ≤4,010,939 KRW (2971 USD) | 70% |
| 4 | ≤5,156,922 KRW (3820 USD) | 90% |
| 5 | ≤5,729,913 KRW (4244 USD) | 100% |
| 6 | ≤7,448,887 KRW (5518 USD) | 130% |
| 7 | ≤8,594,870 KRW (6367 USD) | 150% |
| 8 | ≤11,459,826 KRW (8489 USD) | 200% |
| 9 | ≤17,189,739 KRW (12,733 USD) | 300% |
| 10 | >17,189,739 KRW (12,733 USD) | - |

KRW, South Korean won; USD, United States Dollar.

**Table S2**. Comparison of the weighted average precision (PPV), weighted average F1-score, and AUPRC of the prediction models on the train and test data set (discovery cohort) and the external validation data set (validation cohort).

| Model | Precision | F1-score | AUPRC |
| --- | --- | --- | --- |
| **Train data set (discovery cohort; 5-fold cross-validation)** |  |  |  |
| XGBoost, mean (SD) | 0.9531 (0.0018) | 0.9472 (0.0023) | 0.6483 (0.0124) |
| Random Forest, mean (SD) | 0.9458 (0.0059) | 0.8912 (0.0059) | 0.6401 (0.0117) |
| Light GBM, mean (SD) | 0.9468 (0.0015) | 0.8324 (0.0025) | 0.6880 (0.0113) |
| GBM, mean (SD) | 0.9463 (0.0015) | 0.8654 (0.0023) | 0.6598 (0.0131) |
| AdaBoost, mean (SD) | 0.9486 (0.0014) | 0.8382 (0.0023) | 0.7155 (0.0077) |
| Logistic regression, mean (SD) | 0.9468 (0.0015) | 0.8586 (0.0007) | 0.4155 (0.0049) |
| GBM & AdaBoost, mean (SD) | 0.9477 (0.0013) | 0.8550 (0.0042) | 0.6965 (0.0114) |
| AdaBoost & LR, mean (SD) | 0.9477 (0.0014) | 0.8652 (0.0005) | 0.6532 (0.0120) |
| LR & GBM, mean (SD) | 0.9475 (0.0014) | 0.8505 (0.0007) | 0.6389 (0.0113) |
| GBM & AdaBoost & LR, mean (SD) | 0.9480 (0.0015) | 0.8422 (0.0006) | 0.6825 (0.0120) |
| **Test data set (discovery cohort)** |  |  |  |
| XGBoost | 0.9546 | 0.9497 | 0.6616 |
| Random Forest | 0.9476 | 0.7505 | 0.7364 |
| Light GBM | 0.9468 | 0.8361 | 0.7141 |
| GBM | 0.9476 | 0.8771 | 0.6826 |
| AdaBoost | 0.9491 | 0.8355 | 0.7145 |
| LR | 0.9475 | 0.8584 | 0.4211 |
| GBM & AdaBoost | 0.9488 | 0.8481 | 0.7000 |
| AdaBoost & LR | 0.9483 | 0.8649 | 0.6554 |
| LR & GBM | 0.9486 | 0.8544 | 0.6465 |
| GBM & AdaBoost & LR | 0.9489 | 0.8455 | 0.6854 |
| **External validation data set (validation cohort)** |  |  |  |
| XGBoost | 0.9256 | 0.8307 | 0.3919 |
| Random Forest | 0.9270 | 0.7619 | 0.5313 |
| Light GBM | 0.9229 | 0.8541 | 0.4671 |
| GBM | 0.9252 | 0.8902 | 0.4248 |
| AdaBoost | 0.9305 | 0.8392 | 0.4701 |
| LR | 0.9309 | 0.8656 | 0.2976 |
| GBM & AdaBoost | 0.9273 | 0.8630 | 0.4510 |
| AdaBoost & LR | 0.9316 | 0.8720 | 0.4434 |
| LR & GBM | 0.9315 | 0.8675 | 0.4306 |
| GBM & AdaBoost & LR | 0.9315 | 0.8769 | 0.4603 |

AdaBoost, Adaptive Boosting; AUPRC, Area Under the Precision-Recall Curve; GBM, Gradient Boosting Machine; LR, logistic regression; SD, standard deviation; XGBoost, eXtreme Gradient Boosting.

**Table S3.** Ablation study results.

| Model | Sensitivity, mean (SD) | Specificity, mean (SD) | Accuracy, mean (SD) | Balanced accuracy, mean (SD) | AUROC, mean (SD) |
| --- | --- | --- | --- | --- | --- |
| All | 0.8062  (0.0072) | 0.8179  (0.0015) | 0.8173  (0.0012) | 0.8120  (0.0030) | 0.9012  (0.0046) |
| Excluding age | 0.7589  (0.013) | 0.8045  (0.0015) | 0.8022  (0.0011) | 0.7817  (0.0060) | 0.8636  (0.0071) |
| Excluding DBP | 0.7831  (0.0086) | 0.7850  (0.0019) | 0.7849  (0.0017) | 0.7840  (0.0041) | 0.8707  (0.0038) |
| Excluding body mass index | 0.8048  (0.0096) | 0.8174  (0.0020) | 0.8167  (0.0016) | 0.8111  (0.0043) | 0.8981  (0.0050) |
| Excluding SBP | 0.7935  (0.013) | 0.8077  (0.0021) | 0.8070  (0.0019) | 0.8006  (0.0061) | 0.8902  (0.0049) |
| Excluding fasting blood glucose | 0.8060  (0.0077) | 0.8177  (0.0016) | 0.8171  (0.0013) | 0.8118  (0.0034) | 0.9010  (0.0046) |
| Excluding DBP and SBP | 0.7369  (0.011) | 0.7127  (0.0010) | 0.7139  (0.0011) | 0.7248  (0.0056) | 0.7966  (0.0039) |

AUROC, Area Under Receiver Operating Characteristic; DBP, diastolic blood pressure; SBP, systolic blood pressure; SD, standard deviation.

**Table S4**. ORs (95% CI) of 5-year incidence of hypertension for each risk factor in the discovery cohort (Korea).

| Risk factors | Events (%) | OR (95% CI) | |
| --- | --- | --- | --- |
|  |  | Univariable | Multivariable^*^ |
| **Sex** |  |  |  |
| Male | 10,129 (4.44) | 1.0 (reference) | 1.0 (reference) |
| Female | 12,093 (5.57) | **1.27 (1.24-1.30)** | **1.14 (1.02-1.27)** |
| **Age** |  |  |  |
| 20–39 y | 1381 (0.91) | 1.0 (reference) | 1.0 (reference) |
| 40–59 y | 11,498 (5.06) | **5.82 (5.50-6.15)** | **4.64 (4.37-4.92)** |
| ≥60 y | 9343 (14.27) | **18.17 (17.16-19.24)** | **11.72 (11.02-12.47)** |
| **Region of residence** |  |  |  |
| Urban | 12,983 (5.35) | 1.0 (reference) | 1.0 (reference) |
| Rural | 9239 (4.57) | **0.85 (0.82-0.87)** | **0.86 (0.83-0.88)** |
| **Household income** |  |  |  |
| Low (0th–33th percentile) | 6826 (5.08) | 1.0 (reference) | 1.0 (reference) |
| Middle (34th–66th percentile) | 6210 (4.30) | **1.19 (1.15-1.23)** | **1.07 (1.03-1.11)** |
| High (67th–100th percentile) | 9186 (5.53) | **1.30 (1.26-1.34)** | **1.26 (1.22-1.31)** |
| **Blood pressure** |  |  |  |
| SBP <130 mmHg and DBP <80 mmHg | 5633 (1.68) | 1.0 (reference) | 1.0 (reference) |
| SBP ≥130mmHg or DBP ≥80 mmHg | 16,589 (15.05) | **10.35 (10.03-10.68)** | **8.31 (8.05-8.58)** |
| **Fasting blood glucose** |  |  |  |
| <100 mg/dL | 14,746 (4.12) | 1.0 (reference) | 1.0 (reference) |
| ≥100 mg/dL | 7476 (8.58) | **0.46 (0.45-0.47)** | **0.83 (0.80-0.86)** |
| **Serum total cholesterol** |  |  |  |
| <200 mg/dL | 11,500 (3.86) | 1.0 (reference) | 1.0 (reference) |
| 200 to 239 mg/dL | 7592 (6.70) | **1.79 (1.74-1.84)** | **1.17 (1.13-1.21)** |
| ≥240 mg/dL | 3130 (9.32) | **2.56 (2.46-2.67)** | **1.27 (1.21-1.33)** |
| **Hemoglobin, g/dL** |  |  |  |
| Low (0th–33th percentile) | 6561 (4.39) | 1.0 (reference) | 1.0 (reference) |
| Middle (34th–66th percentile) | 7567 (5.16) | **1.19 (1.15-1.23)** | 1.00 (0.96-1.04) |
| High (67th–100th percentile) | 8094 (5.44) | **1.25 (1.21-1.30)** | **1.06 (1.01-1.12)** |
| **Aspartate transaminase, U/L** |  |  |  |
| Low (0th–33th percentile) | 4187 (3.03) | 1.0 (reference) | 1.0 (reference) |
| Middle (34th–66th percentile) | 7893 (4.68) | **1.57 (1.51-1.63)** | 1.02 (0.98-1.07) |
| High (67th–100th percentile) | 10,142 (7.33) | **2.53 (2.44-2.62)** | **1.15 (1.09-1.21)** |
| **Alanine transaminase, U/L** |  |  |  |
| Low (0th–33th percentile) | 4161 (2.91) | 1.0 (reference) | 1.0 (reference) |
| Middle (34th–66th percentile) | 7653 (4.93) | **1.73 (1.67-1.80)** | 0.98 (0.93-1.02) |
| High (67th–100th percentile) | 10,408 (7.10) | **2.55 (2.46-2.65)** | **2.05 (2.00-2.11)** |
| **γ-glutamyl transpeptidase, U/L** |  |  |  |
| Low (0th–33th percentile) | 4825 (3.06) | 1.0 (reference) | 1.0 (reference) |
| Middle (34th–66th percentile) | 6660 (4.66) | **1.55 (1.49-1.61)** | **1.12 (1.07-1.16)** |
| High (67th–100th percentile) | 10,737 (7.45) | **2.55 (2.46-2.64)** | **2.37 (2.31-2.44)** |
| **Body mass index** |  |  |  |
| Underweight (<18.5 kg/m2) | 489 (1.70) | 1.0 (reference) | 1.0 (reference) |
| Normal (18.5–22.9 kg/m2) | 6997 (3.11) | **1.86 (1.70-2.04)** | **1.20 (1.09-1.33)** |
| Overweight (23.0–24.9 kg/m2) | 5989 (5.92) | **3.64 (3.32-4.00)** | **2.60 (2.45-2.77)** |
| Obese (≥25.0 kg/m2) | 8747 (9.68) | **6.20 (5.65-6.80)** | **4.31 (4.09-4.55)** |
| **History of diabetes mellitus** |  |  |  |
| No | 21,015 (4.80) | 1.0 (reference) | 1.0 (reference) |
| Yes | 1207 (16.49) | **3.92 (3.68-4.17)** | **3.70 (3.57-3.84)** |
| **History of stroke** |  |  |  |
| No | 22,138 (4.98) | 1.0 (reference) | 1.0 (reference) |
| Yes | 84 (14.95) | **3.35 (2.66-4.23)** | **3.17 (2.89-3.53)** |
| **Smoking** |  |  |  |
| Non-smoker | 16,082 (5.00) | 1.0 (reference) | 1.0 (reference) |
| Ex-smoker | 1045 (5.55) | **1.12 (1.05-1.19)** | **1.09 (1.05-1.15)** |
| Current smoker | 5095 (4.89) | 0.98 (0.95-1.01) | 0.98 (0.94-1.02) |
| **Alcohol intake** |  |  |  |
| <1 day/week | 16,072 (4.81) | 1.0 (reference) | 1.0 (reference) |
| 1–2 day/week | 3845 (4.65) | 0.97 (0.93-1.00) | **1.07 (1.02-1.11)** |
| 3–4 day/week | 1458 (7.11) | **1.52 (1.43-1.60)** | **1.22 (1.14-1.30)** |
| ≥5 day/week | 847 (11.40) | **2.55 (2.37-2.74)** | **1.38 (1.27-1.50)** |
| **Physical activity** |  |  |  |
| Insufficient | 17,798 (4.79) | 1.0 (reference) | 1.0 (reference) |
| Sufficient | 4424 (6.01) | **1.27 (1.23-1.31)** | 0.97 (0.94-1.01) |

CI, confidence interval; DBP, diastolic blood pressure; OR, odds ratio; SBP, systolic blood pressure.

The data in bold indicate significant differences (P < .05).

* Multivariable analysis was adjusted with age, sex, region of residence, household income, blood pressure, fasting blood glucose, serum total cholesterol, hemoglobin, aspartate transaminase, alanine transaminase, γ-glutamyl transpeptidase, body mass index, history of diabetes mellitus, history of stroke, smoking, alcohol intake, and physical activity.

**Table S5**. ORs (95% CI) of 5-year incidence of hypertension for each risk factor in the validation cohort (Japan).

| Risk factors | Events (%) | OR (95% CI) | |
| --- | --- | --- | --- |
|  |  | Univariable | Multivariable^*^ |
| **Sex** |  |  |  |
| Male | 60,346 (4.66) | 1.0 (reference) | 1.0 (reference) |
| Female | 114,756 (6.23) | **1.36 (1.35-1.37)** | **1.28 (1.27-1.29)** |
| **Age** |  |  |  |
| 20–39 y | 17,577 (1.82) | 1.0 (reference) | 1.0 (reference) |
| 40–59 y | 133,330 (6.69) | **3.86 (3.80-3.92)** | **3.22 (3.17-3.28)** |
| ≥60 y | 24,195 (13.54) | **8.43 (8.26-8.60)** | **5.67 (5.54-5.80)** |
| **Blood pressure** |  |  |  |
| SBP <130 mmHg and DBP <80 mmHg | 68,763 (2.91) | 1.0 (reference) | 1.0 (reference) |
| SBP ≥130mmHg or DBP ≥80 mmHg | 106,339 (13.83) | **5.36 (5.31-5.41)** | **4.35 (4.30-4.39)** |
| **Fasting blood glucose** |  |  |  |
| <100 mg/dL | 121,751 (4.61) | 1.0 (reference) | 1.0 (reference) |
| ≥100 mg/dL | 53,351 (10.78) | **2.50 (2.47-2.53)** | **1.38 (1.36-1.39)** |
| **Serum total cholesterol** |  |  |  |
| <200 mg/dL | 69,044 (4.34) | 1.0 (reference) | 1.0 (reference) |
| 200 to 239 mg/dL | 70,482 (6.29) | **1.48 (1.46-1.49)** | **1.05 (1.04-1.06)** |
| ≥240 mg/dL | 35,576 (8.40) | **2.02 (1.99-2.05)** | **1.15 (1.14-1.17)** |
| **Hemoglobin, g/dL** |  |  |  |
| Low (0th–33th percentile) | 47,058 (4.39) | 1.0 (reference) | 1.0 (reference) |
| Middle (34th–66th percentile) | 56,376 (5.56) | **1.28 (1.27-1.30)** | 1.00 (0.98-1.01) |
| High (67th–100th percentile) | 71,668 (6.83) | **1.60 (1.58-1.62)** | **1.08 (1.06-1.10)** |
| **Aspartate transaminase, U/L** |  |  |  |
| Low (0th–33th percentile) | 46,365 (4.15) | 1.0 (reference) | 1.0 (reference) |
| Middle (34th–66th percentile) | 51,958 (5.17) | **1.26 (1.24-1.28)** | 1.00 (0.98-1.01) |
| High (67th–100th percentile) | 76,779 (7.59) | **1.90 (1.88-1.92)** | **1.12 (1.10-1.14)** |
| **Alanine transaminase, U/L** |  |  |  |
| Low (0th–33th percentile) | 36,258 (3.71) | 1.0 (reference) | 1.0 (reference) |
| Middle (34th–66th percentile) | 55,958 (5.16) | **1.41 (1.39-1.43)** | **0.94 (0.93-0.96)** |
| High (67th–100th percentile) | 82,886 (7.73) | **2.18 (2.15-2.21)** | 0.97 (0.96-1.00) |
| **γ-glutamyl transpeptidase, U/L** |  |  |  |
| Low (0th–33th percentile) | 33,138 (3.31) | 1.0 (reference) | 1.0 (reference) |
| Middle (34th–66th percentile) | 52,647 (4.85) | **1.49 (1.47-1.51)** | **1.13 (1.11-1.15)** |
| High (67th–100th percentile) | 89,317 (8.52) | **2.73 (2.69-2.76)** | **1.43 (1.41-1.46)** |
| **Body mass index** |  |  |  |
| Underweight (<18.5 kg/m2) | 8309 (2.62) | 1.0 (reference) | 1.0 (reference) |
| Normal (18.5–22.9 kg/m2) | 70,538 (4.03) | **1.56 (1.52-1.60)** | **1.25 (1.22-1.28)** |
| Overweight (23.0–24.9 kg/m2) | 40,685 (7.05) | **2.82 (2.75-2.88)** | **1.72 (1.68-1.77)** |
| Obese (≥25.0 kg/m2) | 55,570 (11.32) | **4.74 (4.63-4.85)** | **2.46 (2.40-2.52)** |
| **History of diabetes mellitus** |  |  |  |
| No | 167,497 (5.41) | 1.0 (reference) | 1.0 (reference) |
| Yes | 7605 (20.47) | **4.50 (4.39-4.62)** | **2.92 (2.86-2.98)** |
| **History of stroke** |  |  |  |
| No | 173,706 (5.56) | 1.0 (reference) | 1.0 (reference) |
| Yes | 1396 (16.36) | **3.33 (3.14-3.52)** | **2.36 (2.21-2.52)** |
| **Smoking** |  |  |  |
| No | 121,839 (5.13) | 1.0 (reference) | 1.0 (reference) |
| Yes | 53,263 (6.99) | **1.39 (1.38-1.41)** | **1.24 (1.22-1.25)** |
| **Alcohol intake** |  |  |  |
| Rarely | 80,223 (4.96) | 1.0 (reference) | 1.0 (reference) |
| Sometimes | 51,537 (5.29) | **1.07 (1.06-1.08)** | **1.03 (1.02-1.04)** |
| Every day | 43,342 (7.97) | **1.66 (1.64-1.68)** | **1.22 (1.20-1.23)** |
| **Physical activity** |  |  |  |
| Insufficient | 139,826 (5.40) | 1.0 (reference) | 1.0 (reference) |
| Sufficient | 35,276 (6.46) | **1.21 (1.20-1.22)** | 0.98 (0.95-1.00) |

CI, confidence interval; DBP, diastolic blood pressure; OR, odds ratio; SBP, systolic blood pressure.

The data in bold indicate significant differences (P < .05).

* Multivariable analysis was adjusted with age, sex, blood pressure, fasting blood glucose, serum total cholesterol, hemoglobin, aspartate transaminase, alanine transaminase, γ-glutamyl transpeptidase, body mass index, history of diabetes mellitus, history of stroke, smoking, alcohol intake, and physical activity.
