# Supplementary material for: Machine Learning–Based Prediction for Incident Hypertension Based on Regular Health Checkup Data: Derivation and Validation in 2 Independent Nationwide Cohorts in South Korea and Japan
Source: J Med Internet Res. 2024 Nov 5;26:e52794. doi: 10.2196/52794 (PMC11576616; doi:10.2196/52794)
Supplement: Multimedia Appendix 2 [file jmir_v26i1e52794_app2.docx]

**Figure S1**. Deployed web application to provide hypertension onset within 5 years: (a) a user's web interface to enter information, (b) the prediction results with the probability.


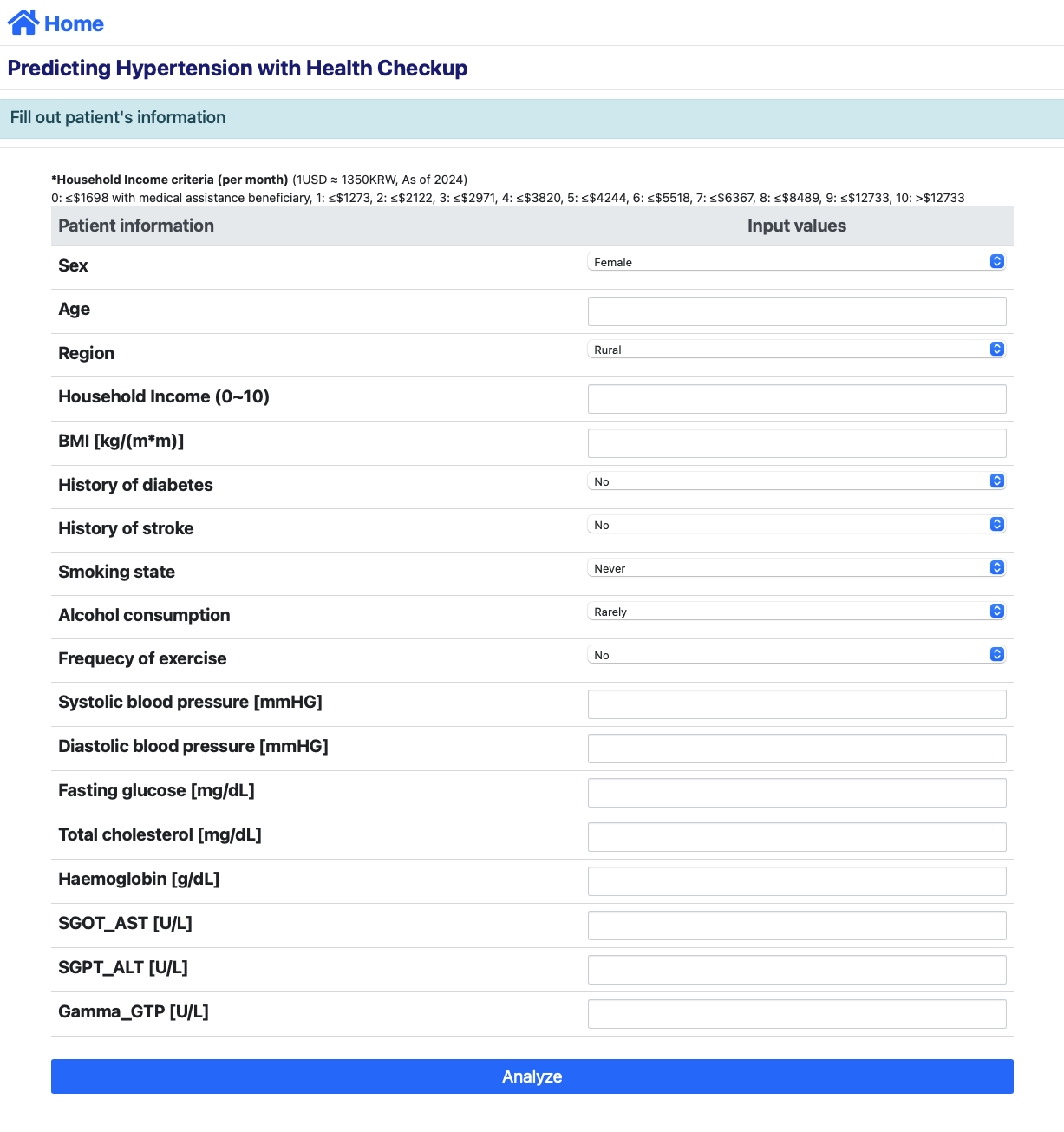


(a)


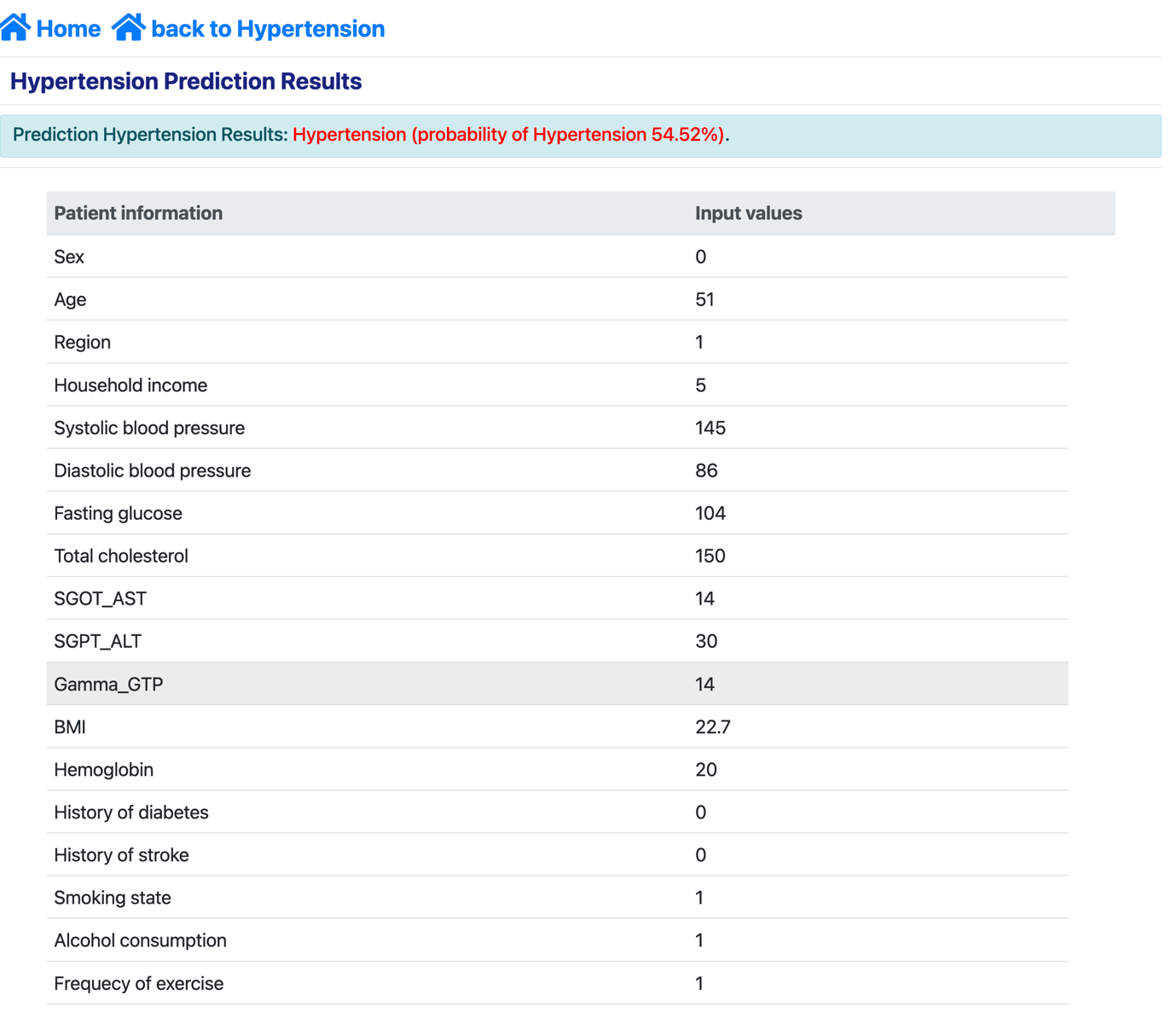


(b)
